# Mutation of the rice XA21 predicted nuclear localization sequence does not affect resistance to *Xanthomonas oryzae* pv. *oryzae*

**DOI:** 10.7717/peerj.2507

**Published:** 2016-10-05

**Authors:** Tong Wei, Tsung-Chi Chen, Yuen Ting Ho, Pamela C. Ronald

**Affiliations:** 1 Department of Plant Pathology and the Genome Center, University of California, Davis, CA, United States; 2 Feedstocks Division, Joint BioEnergy Institute, Lawrence Berkeley National Laboratory, Berkeley, CA, United States; 3 Biological Systems and Engineering Division, Lawrence Berkeley National Laboratory, Berkeley, CA, United States

**Keywords:** Nuclear localization, Bacterial blight disease, *Xanthomonas oryzae* pv. *oryzae*, XA21-mediated immunity

## Abstract

**Background:**

The rice receptor kinase XA21 confers robust resistance to the bacterial pathogen *Xanthomonas oryzae*pv. *oryzae*(*Xoo*). We previously reported that XA21 is cleaved in transgenic plants overexpressing XA21 with a GFP tag (*Ubi*-XA21-GFP) and that the released C-terminal domain is localized to the nucleus. XA21 carries a predicted nuclear localization sequence (NLS) that directs the C-terminal domain to the nucleus in transient assays, whereas alanine substitutions in the NLS disrupt the nuclear localization.

**Methods:**

To determine if the predicted NLS is required for XA21-mediated immunity *in planta*, we generated transgenic plants overexpressing an XA21 variant carrying the NLS with the same alanine substitutions (*Ubi*-XA21nls-GFP).

**Results:**

*Ubi-*XA21nls-GFP plants displayed slightly longer lesion lengths, higher *Xoo*bacterial populations after inoculation and lower levels of reactive oxygen species production compared with the *Ubi-*XA21-GFP control plants. However, the *Ubi-*XA21nls-GFP plants express lower levels of protein than that observed in *Ubi-*XA21-GFP.

**Discussion:**

These results demonstrate that the predicted NLS is not required for XA21-mediated immunity.

## Introduction

Pattern recognition receptors (PRRs) recognize conserved microbial signatures, activating immune responses (Ronald & Beutler, 2010; Schwessinger & Ronald, 2012). The rice PRR XA21 confers robust resistance to diverse strains of *Xanthomonas oryzae* pv. *oryzae* (*Xoo*), the causal agent of blight disease in rice (Khush, Bacalangco & Ogawa, 1990; Song et al., 1995). XA21 recognizes a tyrosine-sulfated protein called RaxX (required for activation of XA21-mediated immunity X) derived from *Xoo*. Tyrosine sulfation is required for RaxX’s activity. In rice plants expressing XA21, sulfated RaxX activates XA21-mediated immune responses, including production of reactive oxygen species (ROS), ethylene, and induction of defense gene expression (Pruitt et al., 2015).

We previously reported that transgenic plants overexpressing XA21 produce XA21 cleavage products (Chen et al., 2010a; Chen et al., 2010b; Park et al., 2010; Park et al., 2008; Park & Ronald, 2012; Wang et al., 2006). For example, a 110-kDa amino-terminal XA21 cleavage product was detected in *Ubi*-Myc-XA21 transgenic plants and a 70-kDa carboxy-terminal cleavage product was observed in *Ubi*-XA21-CFP and *Ubi*-XA21-GFP plants (Park & Ronald, 2012). A predicted nuclear localization sequence (NLS) located between the XA21 transmembrane and juxtamembrane domain was identified. In rice protoplasts, the predicted NLS is able to direct transiently expressed GFP-tagged XA21 C-terminal domain to the nucleus. In contrast, a construct carrying alanine substitutions in the predicted NLS fails to direct the nuclear localization of XA21 C-terminal domain (Park & Ronald, 2012).

To assess the biological relevance of the predicted XA21 nuclear localization sequence *in planta*, we generated transgenic plants expressing an XA21 variant with alanine substitutions in the predicted NLS (XA21nls). The *Ubi-*XA21nls-GFP transgenic lines displayed partial resistance to *Xoo* infection, as reflected in slightly longer lesion lengths and higher *Xoo* bacterial populations compared with *Ubi-*Xa21-GFP control plants. A tyrosine-sulfated 21-amino acid derivative of RaxX (RaxX21-sY) triggered ROS production in *Ubi-*XA21nls-GFP plants. The expressed protein levels of XA21nls-GFP in *Ubi-*XA21nls-GFP lines were lower than those observed in *Ubi*-XA21-GFP plants. These results suggest that lower levels of expressed protein account for the slightly longer lesions observed in *Ubi-*XA21nls-GFP transgenic plants and indicate that the predicted NLS is not critical for XA21-mediated immunity.

## Materials & Methods

### Rice growth condition and leaf treatment with elicitors

*Oryza sativa* ssp. *japonica* cultivar Kitaake and a transgenic line expressing XA21 (Park & Ronald, 2012), *Ubi*-XA21-GFP (5B-5-4-3-2-1; the transgene driven by the maize *ubiquitin* promoter), were used in this study. The Kitaake genetic background lacks XA21 and is fully susceptible to the *Xoo* strain used in this study. Rice seeds were surfaced-sterilized with 15% bleach, rinsed with water, and soaked in water for one week. Well-germinated seedlings were transplanted into a soil mixture (80% sand, 20% peat from Redi-Gro, Sacramento, CA, USA) in an environmentally-controlled greenhouse.

### Vector construction and rice transformation

The *XA21nls-GFP* (KRTKK678-682AATAA) construct was generated using site-directed mutagenesis. The DNA sequence containing alanine substitutions were amplified from an *XA21-GFP*/pENTR vector (Park & Ronald, 2012) using two overlapping primers (XA21nls-F and XA21nls-R, Table S1). The PCR product was digested with *Dpn*I at 37 °C overnight and transformed into *Escherichia coli* competent cells, followed by kanamycin selection. Plasmids carrying the mutations were confirmed by standard Sanger sequencing. *XA21nls-GFP* was subcloned into *Ubi*-pCAMBIA-1300, which carries the maize ubiquitin promoter to drive expression of the transgene (Chern et al., 2005) using Gateway LR Clonase (Invitrogen, Carlsbad, CA, USA). The resulting *Ubi-XA21nls-GFP* construct was used to transform rice Kitaake calli by *Agrobacterium-*mediated method in Plant Transformation Facility at UC Davis (Hiei et al., 1994). The presence of the transgene was confirmed by PCR using gene-specific primers (Ubi-pro and XA21-seq2 in Table S1).

### Bacterial infection assays

*Xanthomonas oryzae* pv. *oryzae*(*Xoo*) Philippines race 6 PXO99^*A*^ (referred to as PXO99) was used in this study. *Xoo* was cultured on peptone sucrose agar plates supplemented with 20 mg L^−1^ cephalexin for two days, then washed off and re-suspended in sterilized water. The concentration of bacterial suspension was adjusted to an OD_600_ of 0.5 (approximately 1 × 10^8^ cfu/mL) for inoculation.

Before inoculation, 5-week-old greenhouse-grown rice plants were transferred to a walk-in growth chamber (14-h-light/10-h-dark photo-period, 28°C/24 °C, 80%/85% humidity) to acclimate the chamber conditions for one week. Plants were inoculated using the scissor clipping method (Song et al., 1995). For each line, 8-12 plants were inoculated and in each plant two fully-expanded leaves from 3-6 tillers were clipped using scissors with the *Xoo* inoculum. The lesion lengths were measured 14 days post-inoculation. *In planta* bacterial growth was assessed as described previously (Bahar et al., 2014). Briefly, inoculated leaves were collected at indicated time points, cut into 5-mm pieces and incubated in 10 mL sterile water with shaking at 28 °C for 1 h. The suspension was diluted accordingly and spread out on PSA plates with 20 mg L^−1^ cephalexin. The bacterial colonies were counted after a two-day culture at 28 °C.

### Protein extraction and western blot assays

Protein extraction from rice leaves and western blot assays were performed as previously described (Park & Ronald, 2012). Briefly, total protein was extracted from 100 mg of rice leaf tissue in 200 µL of pre-chilled extraction buffer (0.15 M NaCl, 0.01 M sodium phosphate buffer pH 7.2, 2 mM EDTA, 1% Triton X-100, 10 mM DTT, 20 mM NaF, 1 mM PMSF, 1% Sigma protease cocktail) and separated in an 8% SDS-polyacrylamide gel. A mouse anti-GFP antibody (Santa Cruz Biotechnology, Santa Cruz, CA, USA) was used as the primary antibody for detection of GFP-tagged XA21 and XA21nls.

### ROS assays

ROS assays were performed as previously described (Pruitt et al., 2015). Fully-expanded leaves were harvested from 4-week-old hydroponically grown rice plants, cut into 2-mm^2^ pieces and floated on water overnight. Leaf pieces were treated with water, 1 µM nonsulfated 21-amino acid synthetic RaxX peptides (RaxX21-Y) or tyrosine sulfated RaxX21 peptides (RaxX21-sY). For each treatment, four biological replicates were included and in each replicate two leaf pieces were used. Chemiluminescence was recorded every 30 s for 3 h in a high-sensitivity TriStar plate reader (Berthold, Germany).

## Results

### Generation of *Ubi*-XA21nls-GFP transgenic plants

**Figure 1:**
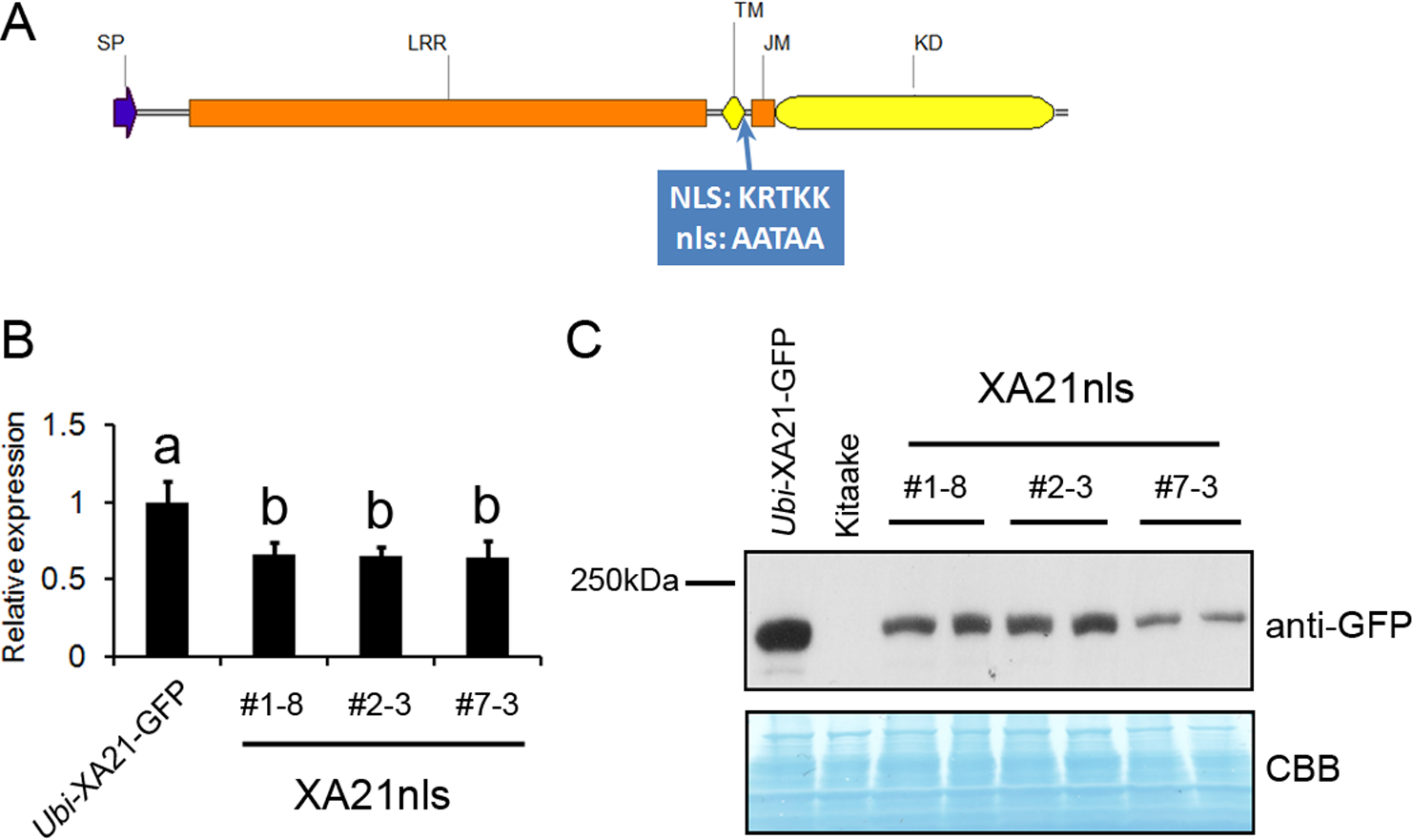
*Ubi*-XA21nls-GFP transgenic plants have reduced transcript and protein levels. (A) Domain organization of XA21 receptor (SP, signal peptide; LRR, leucine rich repeat; TM, transmembrane domain; JM, juxtamembrane domain; KD, kinase domain). The predicted nuclear localization sequence (NLS: KRTKK, amino acids 678-682) highlighted in blue, is mutated to AATAA in *Ubi*-XA21nls-GFP. (B) Relative expression levels of *XA21* in *Ubi*-XA21-GFP and T2 progeny derived from three independent *Ubi*-XA21nls-GFP T1 lines (XA21nls #1–8, #2–3 and #7–3) determined by qRT-PCR and normalized to *ubiquitin* reference gene. The *XA21* expression level in *Ubi-*XA21-GFP was set to 1. Bars represent mean ± SD of three technical replicates. Different letters indicate significant differences between the groups (Tukey’s honestly significant difference test, *α* < 0.05). The experiment was repeated three times with similar results. (C) Western blot analysis of 70 µg total protein extracts from 5-wk *Ubi*-XA21-GFP, Kitaake and two individual T2 plants derived from three *Ubi*-XA21nls-GFP T1 lines. The full-length XA21-GFP was detected with an anti-GFP antibody. Equal loading of total proteins was confirmed by Coomassie blue staining (CBB).

The predicted NLS in XA21 is a basic amino acid-rich sequence localized between the transmembrane and juxtamembrane domains (Park & Ronald, 2012). An XA21 variant with alanine substitutions in the predicted NLS (XA21nls; Fig. 1A) was generated using site-directed mutagenesis and introduced into the rice Kitaake cultivar. Five independent *Ubi*-XA21nls-GFP lines were obtained, and three lines that expressed detectable full-length XA21nls-GFP protein were selected for further analysis. We observed reduced gene and protein expression levels of *XA21-GFP* in these transgenic lines (#1–8, #2–3 and #7–3) as compared with those in the *Ubi-*XA21-GFP control plants (Figs. 1B and 1C).

### The T1 progeny of *Ubi*-XA21nls-GFP plants display resistance to Xoo

To determine whether *Ubi*-XA21nls-GFP confers resistance to *Xoo*, the T1 progeny of three *Ubi*-XA21nls-GFP T0 lines (#1, #2 and #7) were assessed for resistance to *Xoo*. Genotyping revealed that all three *Ubi*-XA21nls-GFP lines segregated for the transgene in the T1 generation (Fig. S1). The ratio of the transgene to null in T1 segregants in line #1, #2 and #7 were 9:6, 2:2 and 8:10 respectively, suggesting the presence of single T-DNA insertions.

The positive segregants in line #1, #2 and #7 (closed columns in Fig. S1) displayed short lesion lengths, while the null segregants (open columns in Fig. S1) displayed lesions as long as the Kitaake control plants. These results demonstrate that the T1 progeny derived from the three *Ubi*-XA21nls-GFP lines displayed similar or slightly longer lesions than the *Ubi*-XA21-GFP control.

### *Ubi*-XA21nls-GFP plants display partial resistance to Xoo

**Figure 2:**
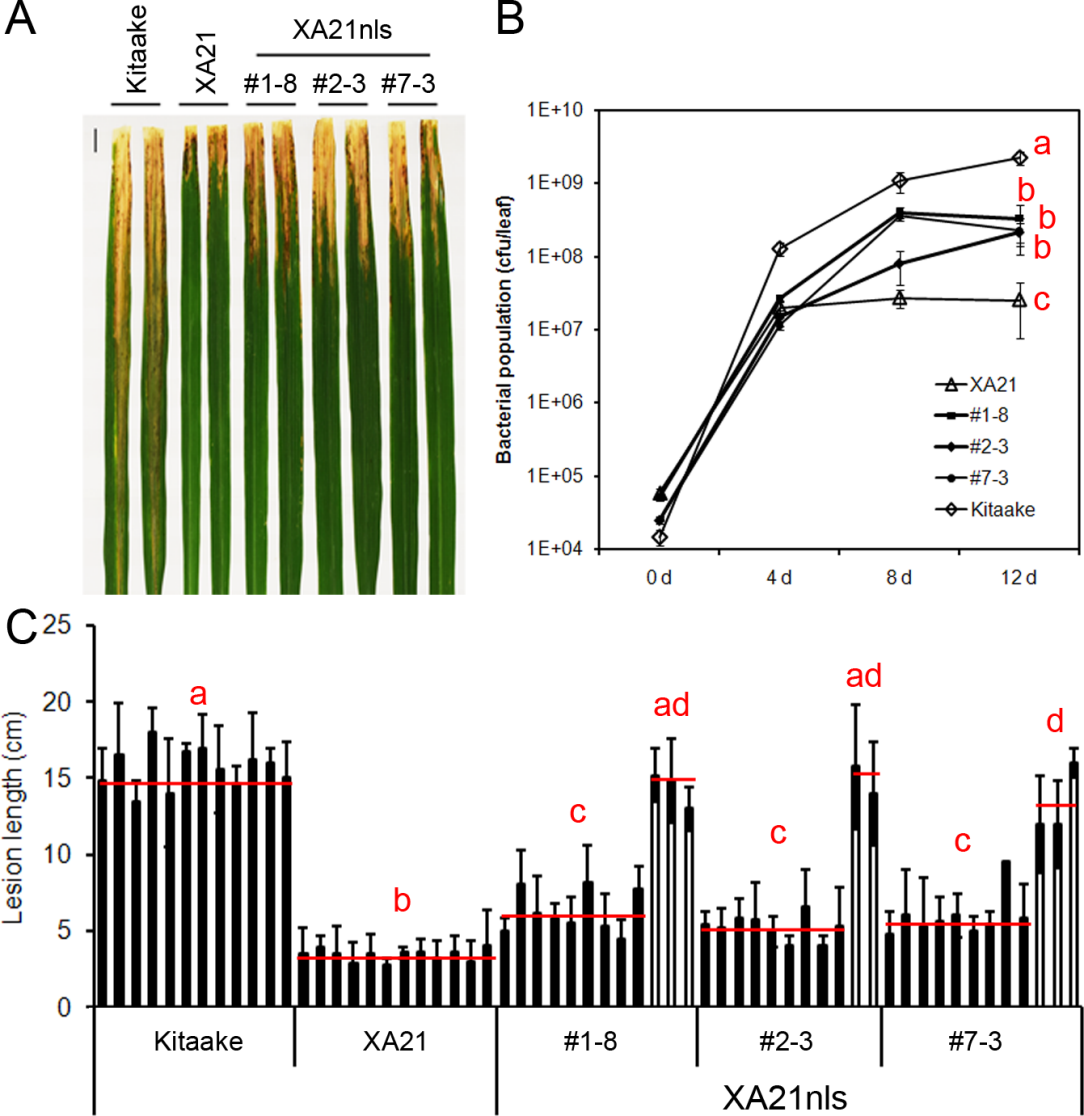
T2 generation of *Ubi*-XA21nls-GFP plants display partial resistance to *Xoo*. (A) Leaves of Kitaake, *Ubi*-XA21-GFP (XA21), and T2 progeny derived from three independent *Ubi*-XA21nls-GFP T1 lines (#1–8, #2–3 and #7–3) 14 days post-inoculation (dpi). (B) Bacterial population in Kitaake, *Ubi*-XA21-GFP, and *Ubi*-XA21nls-GFP plants at 0, 4, 8 and 12 dpi determined by the number of colony-forming units (CFU) per inoculated leaf. Error bars represent standard deviation from at least four leaves. (C) Lesion lengths of Kitaake, *Ubi*-XA21-GFP, and *Ubi*-XA21nls-GFP plants at 14 dpi. The segregants (closed columns) and null segregants (open columns) in each *Ubi*-XA21nls-GFP line were separated into two groups for statistical analysis. Bars represent mean ± SD from about six leaves. The red horizontal line indicates the average lesion length of each genotype. Different letters in (B) and (C) indicate significant differences between the groups (Tukey’s honestly significant difference test, *α* < 0.05). This experiment was repeated a second time (Fig. S2) with similar results.

To validate the observation that the *Ubi*-XA21-GFP plants maintain resistance to *Xoo*, the bacterial infection assays were repeated on the T2 progeny derived from three *Ubi*-XA21nls-GFP transgenic T1 lines (#1–8, #2–3 and #7–3). Five-week-old Kitaake, *Ubi*-XA21-GFP and *Ubi*-XA21nls-GFP plants were inoculated with PXO99. Fourteen days after inoculation, Kitaake plants developed long water-soaked lesions typical of the disease (15.6 ± 2.5 cm), whereas *Ubi*-XA21-GFP developed very short lesions (3.4 ± 1.2 cm) (Figs. 2A and 2C). The *Ubi*-XA21nls-GFP plants segregated for *Xoo* resistance. The null segregants in three *Ubi*-XA21nls-GFP lines displayed long lesions, similar to the susceptible Kitaake plants. In contrast, the positive segregants from these lines displayed relatively short lesions, 6.3 ± 2.1 cm in #1–8, 5.3 ± 1.8 cm in #2–3 and 5.6 ± 1.9 cm in #7–3 (Fig. 2C and Fig. S2). The lesion lengths in the *Ubi*-XA21nls-GFP lines were significantly longer than *Ubi*-XA21-GFP but shorter than Kitaake (Fig. 2C). A second inoculation experiment #2 was performed with additional T2 progeny from the *Ubi*-XA21nls-GFP T1 lines. The slightly longer lesion phenotype also co-segregated with the *Ubi*-XA21nls-GFP transgene (Fig. S2), which is consistent with the results in the first experiment (Fig. 2).

To assess *Xoo* growth *in planta*, the same plants from the first inoculation experiment were used for a bacterial growth assay. Inoculated leaves were collected 0, 4, 8 and 12 days post-inoculation (dpi). As shown in Fig. 2B, the bacterial population in Kitaake reached to 10^8^ cfu/leaf at 4 dpi, whereas the populations in *Ubi*-XA21-GFP and *Ubi*-XA21nls-GFP plants reached over 10^7^ cfu/leaf. At 8 dpi, the *Xoo* population grew in *Ubi*-XA21nls-GFP plants but remained unchanged in the *Ubi*-XA21-GFP control plants. At 12 dpi, the bacterial populations in three *Ubi*-XA21-GFP transgenic lines were significantly higher than the *Ubi-*XA21-GFP plants but lower than that in the Kitaake plants (Fig. 2B). The result of bacterial populations is consistent with the lesion lengths in *Ubi*-XA21nls-GFP plants, indicating that *Ubi*-XA21nls-GFP plants display partial resistance to *Xoo* as compared with the *Ubi-*XA21-GFP control.

### RaxX21-sY triggers ROS production in *Ubi*-XA21nls-GFP plants

**Figure 3:**
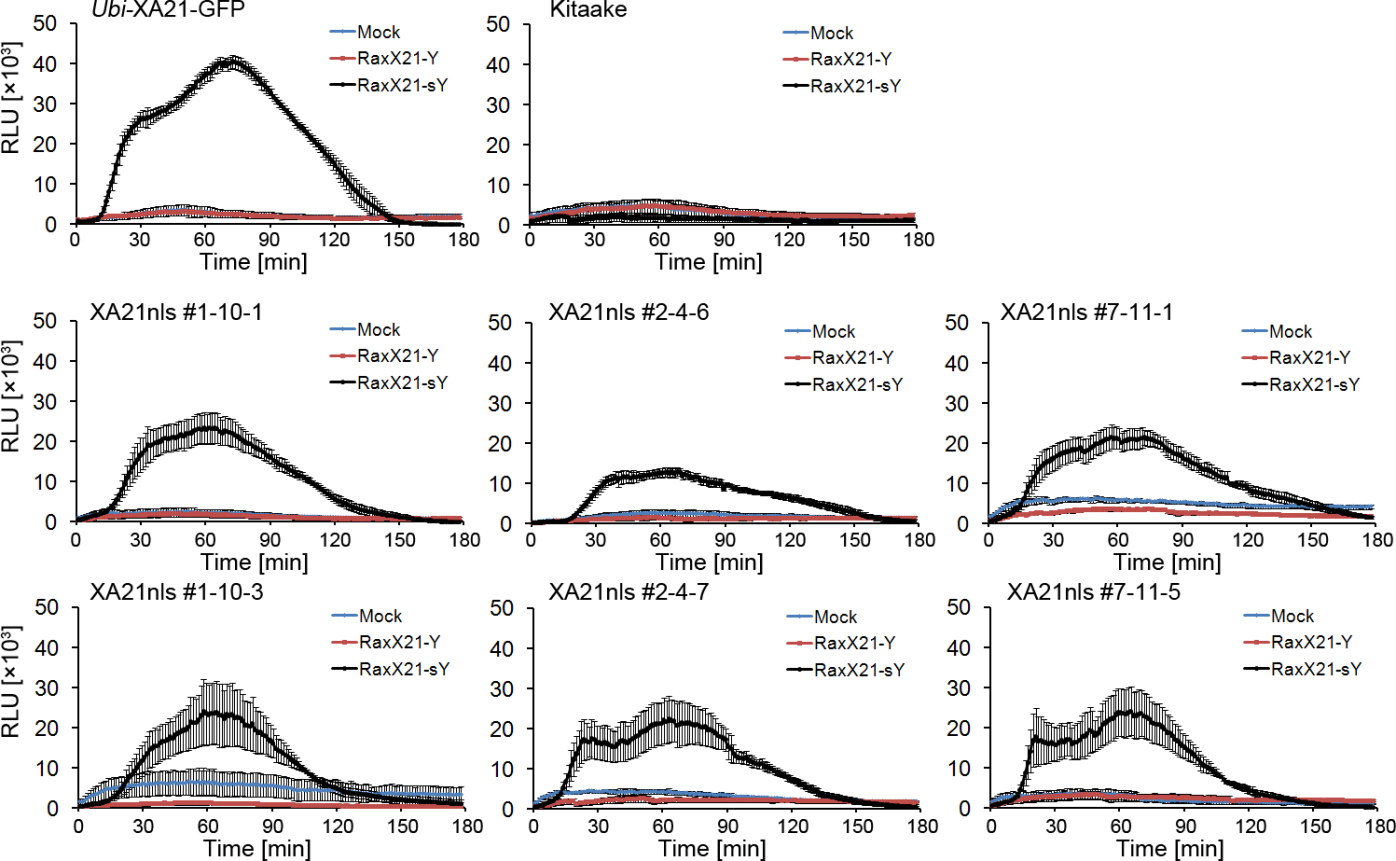
Sulfated RaxX triggers ROS production in *Ubi*-XA21nls-GFP plants. ROS production was measured in leaves from 4-wk hydroponically-grown Kitaake, *Ubi*-XA21-GFP, and T2 transgenic plants derived from three independent *Ubi*-XA21nls-GFP transgenic T1 lines (#1–10, #2–4 and #7–11) treated with water, 1 µM nonsulfated or sulfated 21-amino acid RaxX peptide (Mock, RaxX21-Y and RaxX21-sY, respectively). Bars represent mean ± SE of four technical replicates. RLU: relative light units.

The production of ROS is a hallmark of XA21-mediated immune responses (Pruitt et al., 2015). We therefore carried out a ROS assay to test whether RaxX21-sY treatment leads to ROS production in *Ubi*-XA21nls-GFP plants. Detached leaves from four-week-old hydroponically-grown *Ubi-*XA21-GFP, Kitaake and *Ubi-*XA21nls-GFP plants were treated with water, 1 µM RaxX21-Y or RaxX21-sY. As shown in Fig. 3, RaxX21-sY triggered ROS production in *Ubi-*XA21-GFP plants but not in Kitaake. The ROS production peaked around 70 min and last for 150 min. The three *Ubi-*XA21nls-GFP lines displayed ROS production upon RaxX21-sY treatment, in a similar manner as the *Ubi-*XA21-GFP control plants. RaxX21-sY induced ROS production reached a lower level in three *Ubi-*XA21nls-GFP lines compared with that in *Ubi-*XA21-GFP plants. These results demonstrate that *Ubi-*XA21nls-GFP plants maintain the ROS response to RaxX21-sY.

## Discussion

We previously reported that XA21 is cleaved in transgenic plants overexpressing XA21 with a GFP tag (*Ubi*-XA21-GFP), that the released GFP tagged C-terminal domain is localized to the nucleus and that a predicted NLS directs this domain to the nucleus in transient assays (Park & Ronald, 2012). To investigate the biological relevance of these observations, here we used a genetic approach to assess the resistance of transgenic plants expressing XA21 with mutations in the predicted NLS (*Ubi-*XA21nls-GFP) to *Xoo*.

We observed that the three *Ubi-*XA21nls-GFP transgenic lines displayed slightly reduced resistance to *Xoo*, as illustrated by slightly longer lesion lengths and higher bacterial populations compared with the *Ubi*-XA21-GFP control plants (Fig. 2). Sulfated, but not nonsulfated, RaxX21 is able to trigger ROS production in the *Ubi-*XA21nls-GFP plants, to slightly lower levels than that observed in the *Ubi*-XA21-GFP control (Fig. 3). Considering the relatively lower *XA21* transcript and protein levels of XA21 in *Ubi*-XA21nls-GFP plants as compared with the *Ubi*-XA21-GFP control plants (Figs. 1B and 1C), we hypothesize that the reduced level of XA21 protein accumulation is responsible for the slightly reduced resistance and reduced ROS levels observed in the *Ubi*-XA21nls-GFP plants. The differences in XA21 protein levels in *Ubi*-XA21-GFP vs. *Ubi*-XA21nls-GFP may be due to position effects of the transgene, as we and others have observed in previous studies.

Together, these results indicate that *Ubi-*XA21nls-GFP is able to respond to RaxX21-sY and confers resistance to *Xoo*, which suggest that the predicted NLS is not required for XA21-mediated immunity. This result conflicts with our previous report that disruption of XA21 nuclear localization in *Ubi*-XA21-GFP-NES caused enhanced susceptibility (Park & Ronald, 2012). One possible explanation for this discrepancy is that the addition of the NES to XA21 disrupts activity of XA21.

The biological role of the XA21 cleavage product remains unknown. The XA21 intracellular domain interacts with several proteins predicted to be nuclear localized, including the XB10/WRKY62 transcription factor (Seo et al., 2011). The nuclear localization of the XA21-GFP cleavage products in transient assays suggests a role in transcriptional regulation in the nucleus (Park & Ronald, 2012). However, here we demonstrate that nuclear localization of the XA21 intracellular domain is not critical for XA21-mediated immunity.

##  Supplemental Information

10.7717/peerj.2507/supp-1 Figure S1 T1 generation of *Ubi*-XA21nls-GFP plants display resistance to *Xoo* Lesion lengths and genotyping results of Kitaake, *Ubi*-XA21-GFP (XA21), and T1 progeny derived from three independent *Ubi*-XA21nls-GFP T0 lines (#1, #2 and #7). The inoculation experiments were carried out separately on 8/19/14 (upper panel) and 9/23/14 (lower panel). The genotyping results of *Ubi*-XA21nls-GFP plants were shown in the gel picture under the columns (segregants represented by closed columns and null segregants by open ones). Bars represent mean ± SD from about six leaves in each plant. The statistical analysis was performed using Tukey’s honestly significant difference test to compare the lesion lengths between groups. In the upper panel, Kitaake, null segregants from XA21nls line #1 and #2 are in group “a”, XA21 in “b”, #1 segregants in “c”, and #2 segregants in “bc”; in the lower panel, Kitaake is in group “a”, XA21in “b”, XA21nls#7 segregants in “b”, and null segregants in “c” (*α* < 0.05). The lesion lengths on the T1 progeny from Ubi-XA21nls-GFP line #7 were comparable with those on Ubi-XA21-GFP, due to the Ubi-XA21nls-GFP transgenic plants slightly stressed as reflected in browning at leaf tips.

10.7717/peerj.2507/supp-2 Figure S2 Lesion lengths and genotyping results of Kitaake, *Ubi*-XA21-GFP and T2 progeny derived from three *Ubi*-XA21nls-GFP T1 lines in experiment #2 Leison lengths of Kitaake, *Ubi*-XA21-GFP (XA21) and T2 progeny derived from three *Ubi*-XA21nls-GFP T1 lines (#1–4, #2–1 and #7–10) were measured 14 days post-inoculation. The genotyping results were shown in the gel pictures under the columns (segregants represented by closed columns and null segregants by open ones). Bars represent mean ± SD from about six leaves. The statistical analysis was performed using Tukey’s honestly significant difference test to compare the lesion lengths between groups. Kitaake is in group “a”, XA21 in “b”, *Ubi*-XA21nls-GFP line #1-4 segregants and null segregants in “d” and “ac”, line #2–1 segregants and null segregants in “d” and “c”, and line #7-10 segregants and null segregants in “e” and “ac”, respectively (*α* < 0.05).

10.7717/peerj.2507/supp-3 Table S1 Primers used in this study

## Additional Information and Declarations

### Data Availability

The following information was supplied regarding data availability: The research in this article did not generate, collect or analyse any raw data or code.
