# Supplementary figures and images for: Mutation of the rice XA21 predicted nuclear localization sequence does not affect resistance to Xanthomonas oryzae pv. oryzae
Source: PeerJ. 2016 Oct 5;4:e2507. doi: 10.7717/peerj.2507 (PMC5068440; doi:10.7717/peerj.2507)

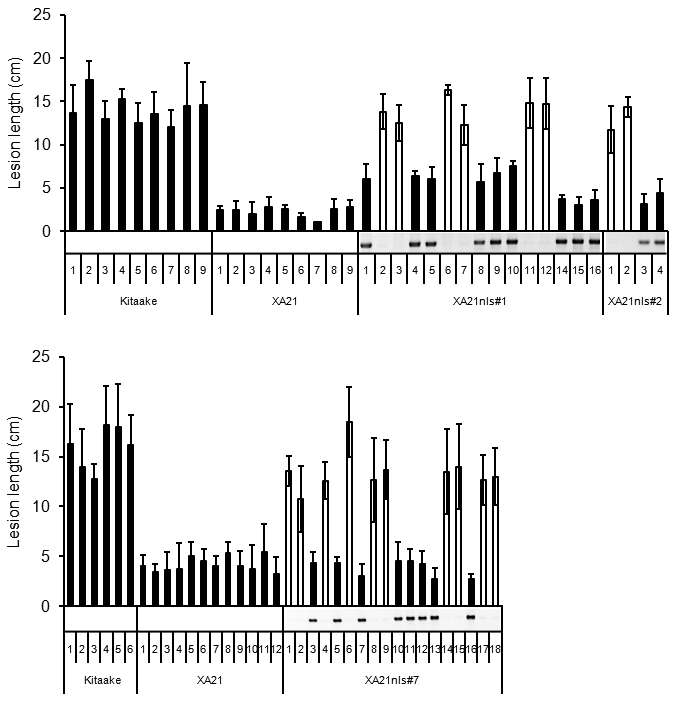

Supplement: Figure S1 — Lesion lengths and genotyping results of Kitaake, Ubi-XA21-GFP (XA21), and T1 progeny derived from three independent Ubi-XA21nls-GFP T0 lines (#1, #2 and #7). The inoculation experiments were carried out separately on 8/19/14 (upper panel) and 9/23/14 (lower panel). The genotyping results of Ubi-XA21nls-GFP plants were shown in the gel picture under the columns (segregants represented by closed columns and null segregants by open ones). Bars represent mean ± SD from about six leaves in each plant. The statistical analysis was performed using Tukey’s honestly significant difference test to compare the lesion lengths between groups. In the upper panel, Kitaake, null segregants from XA21nls line #1 and #2 are in group “a”, XA21 in “b”, #1 segregants in “c”, and #2 segregants in “bc”; in the lower panel, Kitaake is in group “a”, XA21in “b”, XA21nls#7 segregants in “b”, and null segregants in “c” (α < 0.05). The lesion lengths on the T1 progeny from Ubi-XA21nls-GFP line #7 were comparable with those on Ubi-XA21-GFP, due to the Ubi-XA21nls-GFP transgenic plants slightly stressed as reflected in browning at leaf tips. [file peerj-04-2507-s001.png]

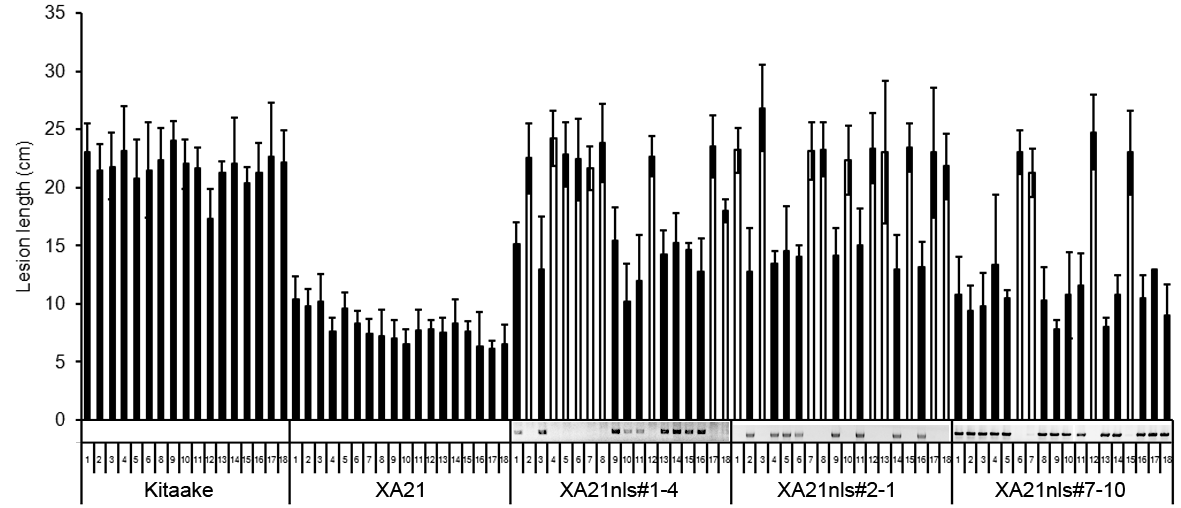

Supplement: Figure S2 — Leison lengths of Kitaake, Ubi-XA21-GFP (XA21) and T2 progeny derived from three Ubi-XA21nls-GFP T1 lines (#1–4, #2–1 and #7–10) were measured 14 days post-inoculation. The genotyping results were shown in the gel pictures under the columns (segregants represented by closed columns and null segregants by open ones). Bars represent mean ± SD from about six leaves. The statistical analysis was performed using Tukey’s honestly significant difference test to compare the lesion lengths between groups. Kitaake is in group “a”, XA21 in “b”, Ubi-XA21nls-GFP line #1-4 segregants and null segregants in “d” and “ac”, line #2–1 segregants and null segregants in “d” and “c”, and line #7-10 segregants and null segregants in “e” and “ac”, respectively (α < 0.05). [file peerj-04-2507-s002.png]
