# Supplementary material for: Mutation of the rice XA21 predicted nuclear localization sequence does not affect resistance to Xanthomonas oryzae pv. oryzae
Source: PeerJ. 2016 Oct 5;4:e2507. doi: 10.7717/peerj.2507 (PMC5068440; doi:10.7717/peerj.2507)
Supplement: Table S1 [file peerj-04-2507-s003.docx]

**Table S1. Primers used in this study.**

| Name | Sequence |
| --- | --- |
| XA21nls-F | TCTACTTGCTTATAACCTGGCACGCGGCAACTGCAGCGGGAGCCCCTTCAAGAACTTC |
| XA21nls-R | GAAGTTCTTGAAGGGGCTCCCGCTGCAGTTGCCGCGTGCCAGGTTATAAGCAAGTAGA |
| Ubi-pro | TTGTCGATGCTCACCCTGTTGTTT |
| XA21-seq2 | ACCACCTAGCTTGTTTTCTCTGAC |
